# Glucosidase II β-subunit, a novel substrate for caspase-3-like activity in rice, plays as a molecular switch between autophagy and programmed cell death

**DOI:** 10.1038/srep31764

**Published:** 2016-08-19

**Authors:** Jing Cui, Bing Chen, Hongjuan Wang, Yue Han, Xi Chen, Wei Zhang

**Affiliations:** 1 Dept. of Biochemistry & Molecular Biology, College of Life Science, Nanjing Agricultural University, Nanjing, Jiangsu 210095, China

## Abstract

Endoplasmic reticulum (ER) stress activates unfolded protein response (UPR) and autophagy. However, prolonged, severe stresses activate programmed cell death (PCD) in both animal and plant cells. Compared to the well-studied UPR pathway, the molecular mechanisms of ER-stress-induced PCD are less understood. Here, we report the identification of Gas2, the glucosidase II β subunit in the ER, as a potential switch between PCD and autophagy in rice. MS analysis identified Gas2, GRP94, and HSP40 protein in a purified caspase-3-like activity from heat stressed rice cell suspensions. The three corresponding genes were down-regulated under DTT-induced ER stress. Gas2 and GRP94 were localized to the ER, while HSP40 localized to the cytoplasm. Compared to wild-type, a *Gas2 RNAi* cell line was much sensitive to DTT treatment and had high levels of autophagy. Both caspase-3 and heat-stressed cell suspension lysate could cleave Gas2, producing a 14 kDa N-terminal fragment. Conditional expression of corresponding C-terminal fragment resulted in enhanced caspase-3-like activity in the protoplasts under heat stress. We proposed that mild ER stress causes down-regulation of Gas2 and induces autophagy, while severe stress results in Gas2 cleavage by caspase-3-like activity and the cleavage product amplifies this activity, possibly participating in the initiation of PCD.

The endoplasmic reticulum (ER) plays an important role in folding, assembling, and secreting proteins in both plant and animal cells. Various environmental factors induce ER stress, which can lead to the accumulation of unfolded/misfolded proteins in the ER. Under normal circumstances, mild ER stress induces the unfolded protein response (UPR) and alleviates ER stress through reducing protein translation, up-regulating chaperones, and degrading misfolded proteins^1^. Recently, it has been demonstrated that autophagy, the major intracellular degradation pathway, can also be induced by ER stress in both plant and animal cells^2^,^3^. Autophagy is involved in a diversity of biological responses in plants—including nutrient starvation^4^,^5^, pathogen infection^6^, abiotic stresses^7^ and senescence^8^—which may result in either cell survival or cell death. ER stress can cause the release of Ca^2+^ from the ER into the cytoplasm through the inhibition or activation of inositol-1,4,5-triphosphate receptor (IP_3_R), and these elevated cytosolic Ca^2+^ levels activate calpain protease and death-associated protein kinase, which participate in initiating autophagy^9^,^10^,^11^. This result suggests Ca^2+^ release from the ER and into the cytoplasm is an important inducer of autophagy. Previously, ER located Bcl-2 has been demonstrated to reduce the release of Ca^2+^ from the ER, thus inhibiting autophagy^12^.

In contrast, severe ER stress results in prolonged accumulation of unfolded and misfolded proteins, eventually leading to programmed cell death (PCD) in both animal and plant cells. In comparison to well-studied death receptor- or mitochondria-mediated PCD, ER stress-mediated PCD is still largely uncharacterized. Three UPR sensors (ATF6, PERK, and IRE1) have been implicated in the initiating ER-induced PCD in animals. For example, upon ER stress PERK and ATF6 up-regulate the expression of CHOP, a transcription factor that inhibits the transcription of Bcl-2 and Bcl-xL, which results in apoptosis induction^13^. IRE1 can interact with TRAF2 and activate the ASK1-JNK complex, the latter of which then transduces the signals in the apoptosis pathway^14^. IRE1 also directly activates caspase-12 in the ER through TRAF2 to induce apoptosis^15^. BI-1, the anti-apoptotic protein, binds to and suppresses the activity of IRE1^16^. Although ER stress-induced PCD is unclear in plants, it has been demonstrated that *Arabidopsis* BI-1 (AtBI-1) is up-regulated by tunicamycin (TM) treatment, thereby inhibiting subsequent PCD^17^. These results indicated that the mechanisms of ER stress-induced PCD may be partially conserved between animal and plant cells.

Recently, a variety of studies have focused on the crosstalk between autophagy and PCD, which indicate that these two processes are highly related, while caspase, the initiator and mediator of PCD, acts as a molecular switch node in this crosstalk. Several studies have identified autophagy-related proteins as substrates for different kinds of caspase^18^,^19^,^20^ and activated caspases can degrade these proteins to influence the autophagic response. Furthermore, the cleavage products of these proteins gain new functions to promote PCD, as illustrated by Beclin 1, a Bcl-2 family member protein, which acts as a pro-autophagic molecule^21^. Under nutrient deprivation and oxidative stress, Beclin 1 is cleaved by caspase-3 and -8, and the cleaved C-terminal fragment amplifies mitochondrion-mediated apoptosis in animal cells^22^. Another example is BAP31, a resident integral protein of the ER membrane. The p20 caspase-8 cleavage fragment of BAP31 directs pro-apoptotic signals between the ER and mitochondria. Adenoviral expression of p20 causes an early release of Ca^2+^ from the ER and concomitant uptake of Ca^2+^ into mitochondria, ultimately amplifying caspase activation and apoptosis through the mitochondrial pathway^23^. These results suggest that there may be cellular molecular switches that control the conversion between the autophagy and PCD under either different stresses or different degrees of a specific stress.

Protein kinase C substrate 80K-H (80K-H), also known as glucosidase II β subunit (Gas2), encodes a soluble protein region that is rich in Glu and Asp residues and contains a putative ER retention signal at the C-terminal region^24^. Gas2 has been identified as a molecule downstream of fibroblast growth factor receptor and keratinocyte growth factor receptor^25^, and recently, Gas2 has been shown to interact with IP_3_R and to regulate IP_3_-induced Ca^2+^-release from the ER into the cytoplasm by interacting with the COOH-terminal tail of IP_3_Rs^26^. In animal cells, deficiencies in Gas2 induce autophagy through the mTOR pathway^27^. In plants, von Numers *et al*.^28^ demonstrated that Gas2 is required for EFR-mediated defense signaling in *Arabidopsis* and that the EFR-controlled responses were reduced or blocked in the T-DNA *Gas2* gene knock-out lines. Since the defense responses in plants are often related to programmed cell death, these results suggested that Gas2 may be involved in plant PCD. In this study, we have demonstrated for the first time that Gas2 is a substrate for caspase-3-like activity in rice and that the cleavage product of Gas2 promoted caspase-3-like activity. On the other hand, deficiency of Gas2 also led to autophagy in rice cell suspensions. These results suggested that Gas2 is an important regulator between autophagy and PCD in rice.

## Results

### Gas2/HSP90/HSP40 formed caspase-3 related protein complex in rice suspension cells under heat stress

**Figure 1:**
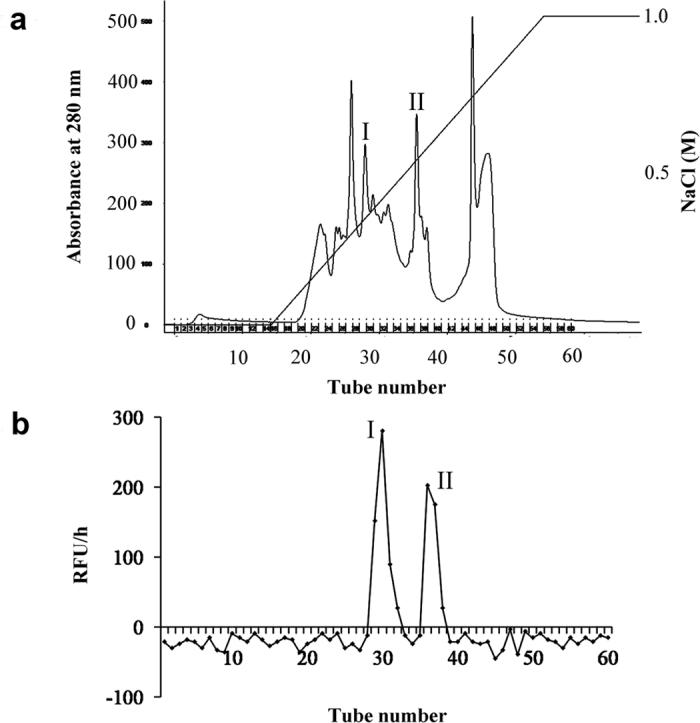
Purification of caspase-3 like activity from heat treated rice suspension cells. (**a**) Caspase-3 active fractions from the G-75 column were pooled and purified further on a Mono-Q column equilibrated with 20 mM Tris/HCl buffer, pH 8.0. Proteins were eluted with a gradient of NaCl (0~1 M) in 20 mM Tris/HCl buffer, pH 8.0, at a flow rate of 1 ml/min. Proteins were detected by the absorbance at 280 nm. Peaks were collected respectively. (**b**) An equal amount of peaks collected from the Mono-Q column were incubated with Ac-DEVD-AMC. The AMC fluorescence was measured by a Microplate reader at 30 °C. RFU/h represented the differences of relative fluorescence units produced in 1h. I and II represent the peaks which exhibited caspase-3 like activity.

Heat stress is a known inducer of caspase-like activities and PCD in *Arabidopsis*^29^, tobacco^30^, and other plant species. Previously, heat treatment has been demonstrated to induce caspase-3-like activity^31^ and PCD in rice suspension cells^32^. In order to identify the putative caspase-3 in rice, caspase-3-like activity was monitored in whole cell lysate and attempts were made to purify this activity by gel filtration, mono-Q hydrophobic chromatography, and biotinyl-DEVD-CHO affinity chromatography. Two elution peaks (I and II) were noted using a NaCl linear gradient (Fig. 1a) that corresponded to significant caspase-3-like activity (Fig. 1b), and these two peaks were identified by LC-MS/MS (Table 1). Three proteins were identified from peak I, which included Gas2, glucose-regulated protein 94 (GRP94), and TPR-containing protein. Additionally, three proteins were identified from peak II, which included methylmalonate-semialdehyde dehydrogenase, peptidyl prolyl cis trans isomerase, and the beta subunit of ATP synthase. Sequence homologue analysis indicated that the TPR-containing protein has a HSP binding motif, STI1, at the C terminus (data not shown), which indicted that this protein likely belongs to the HSP40 family^33^. No caspase-like proteases were identified from peak I and peak II purification, possibly due to the low abundant of this caspase activity. As such, caspase-3-related proteins from peak I were the main focus of the rest of the study.

### Expression profile of *Gas2* under different stresses

**Figure 2:**
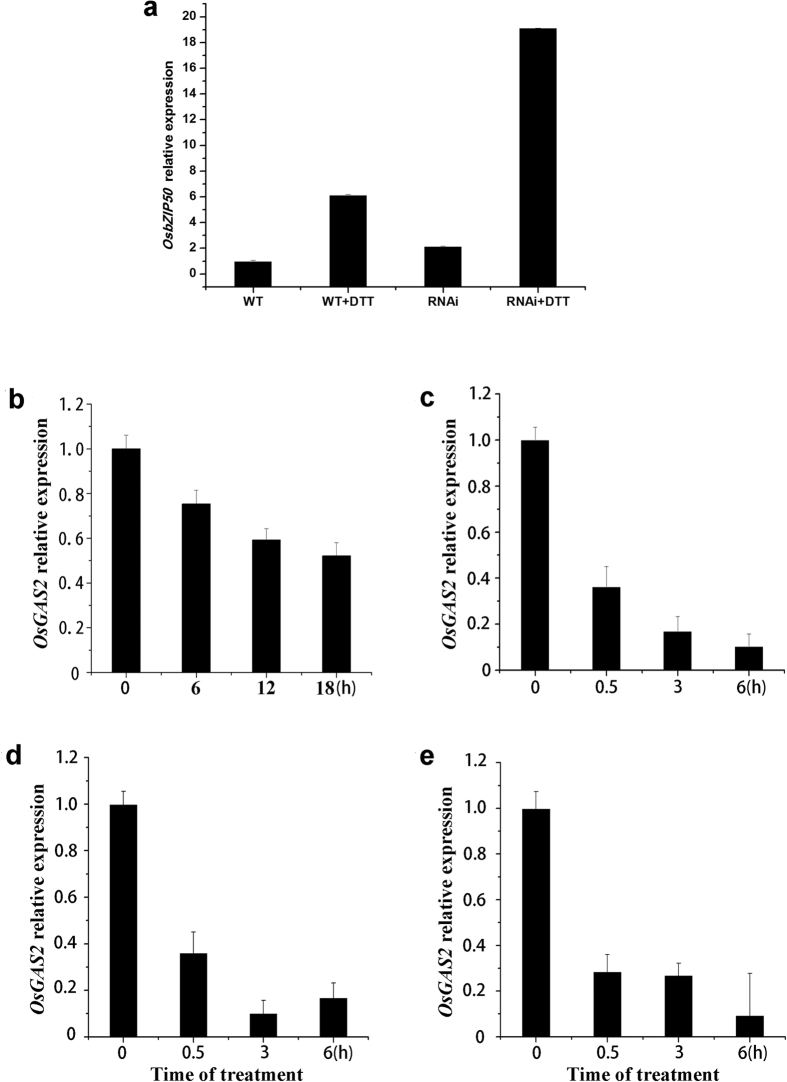
Real-time quantitative RT-PCR analysis of *OsGas2* expression profiles in rice suspension cells under different stresses. (**a**) DTT induced the expression of *OsbZIP50* in wild type and *OsGas2 RNAi* cell lines. Protoplasts were prepared from suspension cells, and treated with 6.4 mM DTT for 18 h at 26–28 °C. (**b**) *OsGAS2* expression in the protoplasts from suspension cells with the treatment of 6.4 mM DTT for different times. (**c–e**) *OsGAS2* expression in 8-day-old seedling roots with the treatment of 4 °C (**c**), 150 mM NaCl (**d**), and 40% PEG 6000 (**e**) for different times. Total RNA was isolated for RT-PCR, and experiments were repeated three times.

Under physiological conditions, Gas2 is a non-catalytic subunit of Glucosidase II, which participates in the glycosylation of the proteins in the ER. In order to identify the biological roles of Gas2 under abiotic stresses, *Gas2* expression was measured by quantitative real-time polymerase chain reaction under several conditions, including under DTT-induced ER stress. Treatment with 6.4 mM DTT induced expression of Os*bZIP50*^34^, the marker gene of UPR, which indicated that DTT was able to induce ER stress in rice suspension cells (Fig. 2a) and, under these conditions, *Gas2* was down-regulated (Fig. 2b). In addition to ER stress, *Gas2* was also down-regulated under different abiotic stresses, such as cold (Fig. 2c), osmotic (Fig. 2d), and salt (Fig. 2e) stresses.

### Sub-cellular localization

**Figure 3:**
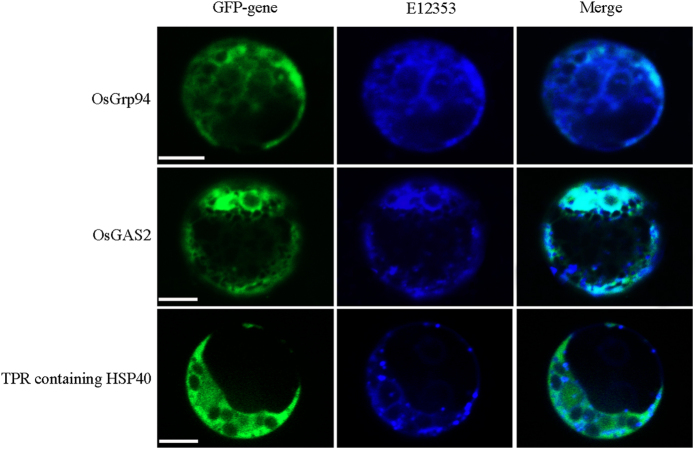
Subcellular localization of Grp94, GAS2 and TPR-containing HSP40 in protoplasts. Constructs carrying 35S:Grp94–GFP, 35S:GAS2–GFP, and 35S:GFA2–GFP, were introduced respectively into protoplasts from the stems of rice seedlings by PEG-calcium-mediated transformation. GFP fluorescence were observed after 16-h incubation with a PerkinElmer UltraVIEW VoX confocal microscope. ER-Tracker™ Blue-White DPX (E12353) was used as an ER localization control. Experiments were repeated at least five times. Bar = 5 μm.

As Gas2 forms a caspase-3 related protein complex with GRP94 and TPR-containing HSP40, the sub-cellular localization of these three proteins was examined. As expected, Gas2 and GRP94 were localized to the ER (Fig. 3a,b), while TPR-containing HSP40 was localized to the cytoplasm (Fig. 3c). Since caspase-3-like activity was present in the cytoplasm, this suggested that under specific stress conditions, TPR-containing HSP40 may form a complex with Gas2 and GRP94 at the interface between ER and cytoplasm, as the ER is known to provide attachment sites for many cytosolic proteins.

### Down-regulation of *Gas2* induced autophagy in rice suspension cell

**Figure 4:**
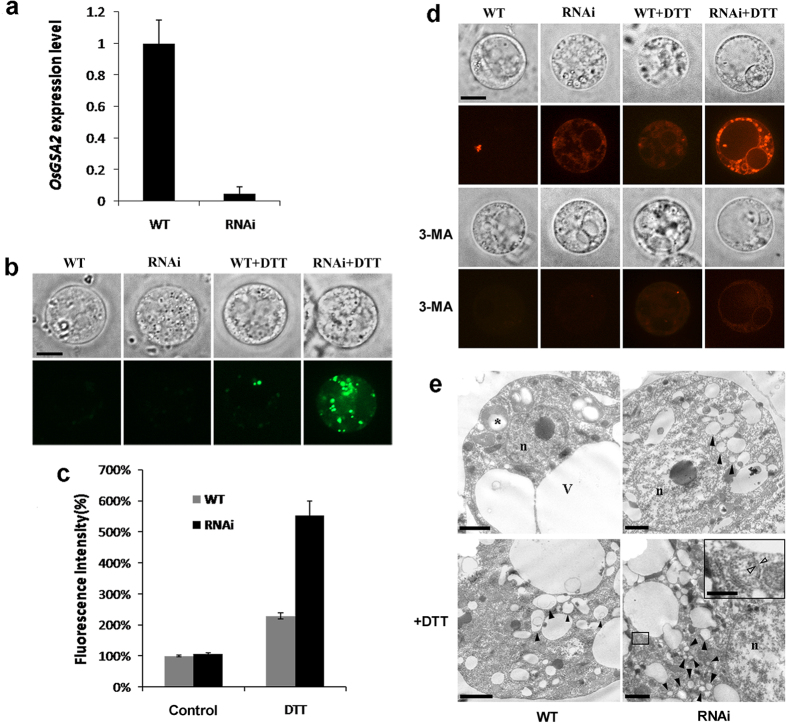
Interference of OsGAS2 expression induced autophagy in rice suspension cells. (**a**) Real-time quantitative RT-PCR analysis of *OsGAS2* expression in wild type and *RNAi* rice suspension cells. (**b**) Cytosolic Ca^2+^ level in wild type and *RNAi* cell lines under DTT treatment. Protoplasts from wild type and *RNAi* lines were treated with 6.4 mM DTT for 18h, then stained with 5 μM FLuo 3-AM, the cytosolic Ca^2+^ probe. protoplasts were imaged with a PerkinElmer UltraVIEW VoX confocal microscope. Bar = 5 μm. (**c**) Quantitative analysis of FLuo 3-AM fluorescence intensity in (**b**). The fluorescence intensity (%) of the protoplasts was measured by delimiting the individual protoplast and the mean fluorescence of the protoplasts was measured. Data represent the mean ± standard error of 8–10 cells. (**d**) Detection of autophagy in wild type and *RNAi* protoplasts. Protoplasts were treated with 6.4 mM DTT with or without 5 mM 3-MA for 18h, and then stained with 50 nm Lytracker red for 20 min at 37 °C. Protoplasts were imaged with a PerkinElmer UltraVIEW VoX confocal microscope. Bar = 5 μm. (**e**) Detection of autophagosomes in wild type and *RNAi* protoplasts. Protoplasts were treated with or without 6.4 mM DTT treatment, and then observed under electron microscope. *starch grains in the plastid. n, nucleus. v, vacuole. black arrowheads indicated numerous vesicles which were accumulated in the cytoplasm. Enlarged inset showed a typical double-membrane bound autophagosomes, and white arrowheads point to the inner and outer membranes of the autophagosome. Bar = 2 μm, and represented 0.5 μm in enlarged inset.

In order to characterize the function of Gas2 in rice, a *Gas2 RNAi* transgenic cell line was constructed, and the expression of *Gas2* was reduced significantly compared to wild-type (Fig. 4a). *Gas2 RNAi* cell line demonstrated normal growth using standard medium (data not shown), however, after DTT treatment, the expression of *OsbZIP50* increased in the *GAS2 RNAi* line compared to the wild-type (Fig. 2a), which indicated that DTT-induced UPR was amplified in the *GAS2 RNAi* line. At the same time, cytosolic Ca^2+^ levels were increased >2-fold in the transgenic lines (Fig. 4b,c). Consistent with noted increased Ca^2+^ levels in the cytoplasm, the autophagosome was observed by LTR staining in transgenic line, but not in wild-type after DTT treatment (Fig. 4d), and this effect could be inhibited by 3-MA, a specific inhibitor of autophagosome formation. Electric microscopy also indicated the formation of double membrane autophagosomes in the *RNAi* line under DTT treatment (Fig. 4e). Taken together, these results indicated that down-regulation of *Gas2* in rice led to higher sensitivity to DTT and accelerated DTT-induced autophagy in rice suspension cells.

### Gas2 is a novel substrate of caspase-3-like activity in rice

**Figure 5:**
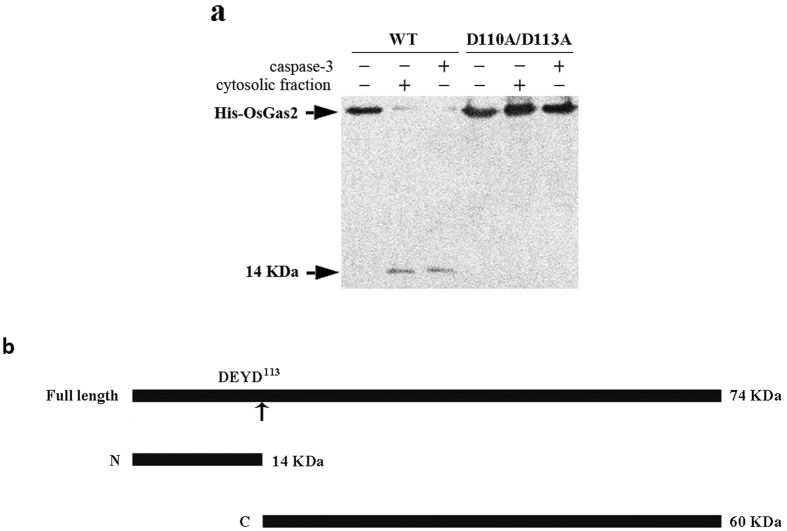
OsGas2 is the substrate of Caspase-3-like activity in rice. (**a**) His_6_-tag was fused at the N-terminus of OsGAS2 and D110A/D113A mutant, and the fusion protein was expressed in *E. coli*. Purified His_6_-tagged wild type and mutant protein were incubated respectively with reaction buffer, caspase-3, and cytosolic faction from heat-treated rice suspension cells. After incubation, the reaction products were analyzed by 12.5% SDS-PAGE followed by immuno-blotting with anti-poly histidine antibody that recognized the N-terminal epitope of His_6_-tagged OsGAS2. Arrows indicated the target protein bands. (**b**) Schematic for OsGas2 fragmentation by caspase-3. The full length OSGas2 with a MW of 74 KDa is illustrated as a linear molecule with a putative caspase-3 cleavage site DEYD^113^, which was indicated by an arrow. Digestion with caspase-3 produced N-terminal fragment with MW of 14 KDa (N), and C-terminal fragment with MW of 60 KDa (**C**).

Gas2/GRP94/HSP40 forms a caspase-3 related complex, which suggests a relationship between Gas2 and caspase-3-like activity. In order to examine this relationship, recombinant OsGas2 was expressed in and purified from *E. coli*. The purified Gas2-His_6_ was incubated with caspase-3 (Sigma-Aldrich) for 5 h at 37 °C and the products were analyzed by immunoblotting using an anti-His-tag antibody. A cleavage product with a MW of 14 kDa could be detected (Fig. 5a), indicating that caspase-3 digested the recombinant OsGas2 and produced a His_6_-tagged 14 kDa N-terminal fragment. In order to verify this result, cytosolic fractions which exhibited caspase-3-like activity were prepared from heat-treated suspension cells. Purified Gas2-His_6_ was incubated with the cytosolic fractions for 5 h at 37 °C and the cleavage products were detected by immunoblotting, as was the 14 kDa cleavage product (Fig. 5a). Consistent with this result, a putative caspase-3 cleavage site (DEYD^113^S) could be found in the OsGas2 sequence, and caspase-3 and cytosolic fractions mentioned above could not cleave the D^110^A/D^113^A mutant (Fig. 5a). These data demonstrate for the first time that OsGas2 is a novel substrate of caspase-3-like activity in rice and that cleavage at D^113^ resulted in a 14 kDa N-terminal fragment and a corresponding C-terminal fragment (Fig. 5b).

### Cleavage product of Gas2 amplified caspase-3-like activity in rice protoplasts

**Figure 6:**
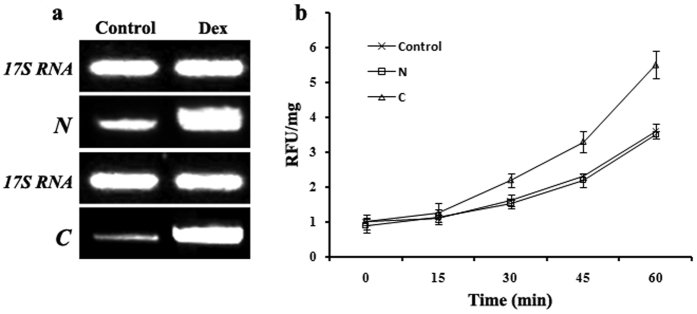
Cleavage product of Gas2 amplified caspase-3-like activity in mesophyll protoplasts. (**a**) RT-PCR analysis of N-terminal fragment (N) and C-terminal fragment (C) expressions in mesophyll protoplasts. Total RNA was prepared from protoplasts with or without (control) treatment of Dex for 18 hour. *17S RNA* was used as a loading control. (**b**) Caspase-3 like activity in mesophyll protoplasts under the expression of N-terminal fragment (N) and C-terminal fragment (C). Expression of N and C were induced by Dex for 18 hours, and then the protoplasts were treated at 48 °C for 15 min and allowed to recover at 28 °C for 6 h. The protoplast with empty conditional expression vector was used as control. An equal amount of cytosolic fractions were incubated with Ac-DEVD-AMC, and the AMC fluorescence was measured within 60 min by a Microplatereader at 30 °C. RFU/mg: relative fluorescence units per mg protein.

In order to identify the possible function of the cleavage products of Gas2, the N- and C-fragments of Gas2 were expressed in rice mesophyll protoplasts using a Dex-induced conditional expression vector and Dex effectively induced the fragment expression (Fig. 6a). Compared to the expression of the N-fragment and vector control, C-fragment expression resulted in a significant increase in caspase-3-like activity after heat treatment (Fig. 6b). Interestingly, no autophagosomes were detected in protoplasts expressing the two fragments after DTT treatment (data not shown).

## Discussion

ER stress is a known inducer of autophagy in animal cells. Elicitors of ER stress, such as TM and DTT, can also induce autophagosome formation in *Arabidopsis*^3^. In this study, DTT treatment was demonstrated to induce *OsbZIP50* expression, the marker gene of ER stress in rice, and a *Gas2 RNAi* cell line was much more sensitive to DTT than wild-type, which suggested that Gas2 might be involved in DTT-induced ER stress in rice suspension cell (Fig. 2). Further, for the first time Gas2 has been implicated in serving a dual function in the crosstalk between autophagy and PCD. As shown in Fig. 4, DTT-induced Ca^2+^ release (Fig. 4b,c) and autophagy (Fig. 4d,e) was more pronounced in *RNAi* line than in the wild-type. Similar to these results, Yang *et al*.^27^ reported that knockdown of *Gas2* in HeLa cell induces autophagy through the mammalian target of rapamycin, although the UPR pathway was not disturbed and no changes in the steady-state concentration of cytosolic Ca^2+^ were observed. These opposing results indicate that, although Gas2 has conserved functions between the animal and plant cells, the molecular mechanisms might be different. On the other hand, it has been reported that Gas2 could interact with IP_3_R, the calcium channel in ER, and participate in the release of Ca^2+^ from ER in animal cells^35^. Although this study did not investigate the relationship between Gas2 and IP_3_R in rice, the results demonstrated that Gas2 participates in the regulation of cytosolic Ca^2+^ concentration in plants.

**Figure 7:**
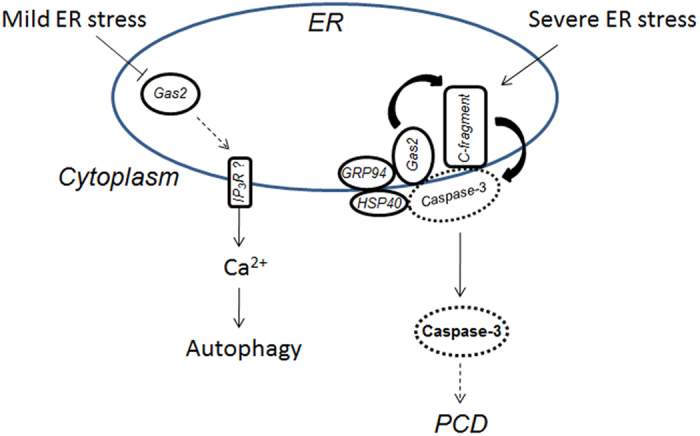
A schematic summary of Gas2 function as a switch between autophagy and PCD in rice. ⊥: inhibition pathway which was demonstrated in this paper; solid arrow: activation, translocation, or degradation pathway which were demonstrated in this paper; dash arrow: the pathway which were not studied in this paper; dash circle: the putative caspase-3-like enzyme. See text for details.

Several caspase-like enzymatic activities have been reported to participate in plant ER stress-induced PCD^36^. In this study, Gas2/GRP94/HSP40 was identified as a caspase-3-related complex, further demonstrating that Gas2 is a novel substrate for caspase-3-like activity. These results suggested that, under severe stress (e.g. heat treatment), heat shock proteins GRP94 and HSP40 provide a processing platform for Gas2, which is then cleaved by caspase-3-like activity at the DEYD^109^S site (Fig. 5). As a result, the C-terminal cleavage product of Gas2 further promotes caspase-3-like activity (Fig. 6b) and forms a caspase-3-amplifying feedback loop. Recently, several caspases were identified as regulators between autophagy and apoptosis in animal cells^37^—caspases can inhibit autophagy by cleaving autophagy-related proteins. After cleavage, the proteins can be converted into pro-apoptotic molecules to induce/promote apoptosis; for example, autophagy-related Atg4D can be cleaved by caspase-3 at the DEVD^63^K site^38^. Overexpression of the C-terminal cleavage products of Atg4D translocated to mitochondria and induced cell death in human cells^38^. Similar with these reports, the C-terminal cleavage product of Gas2 could also amplify the caspase-3-like activity. These results suggest that Gas2 might be a sensor of ER stress in plant cells. As shown in Fig. 7, under mild ER stress, Gas2 was down-regulated, which induce the release of Ca^2+^ from ER to cytoplasm, probably through IP_3_R pathway, and then led to autophagy. Under severe ER stress, HSP40 recruit caspase-3-like activity in cytoplasm, and formed a HSP40/Caspase3/GRP94/Gas2 complex at the interface between ER and cytoplasm. In this complex, caspase-3-like activity cleaved Gas2 at DEYD site. As a product of the cleavage, C-fragment of Gas2 amplified caspase-3 like activity. C-fragment of Gas2 might play partial role in initiating PCD, as DNA fragmentation and cell death was not observed after C-terminal fragment expression, indicating that this amplifying feedback loop is not the only initiator of PCD. As previously mentioned, two caspase-3-related complexes were identified in this study. In addition to Gas2/GRP94/HSP40, FKBP/ATP synthase beta subunit/MMDH was found to form another caspase-3-related complex. In animal cells, the FKBP/ATP synthase subunit has been shown to form PTP transition pores in the inner membrane of mitochondria, which participate in the release of cytochrome *c* and initiate apoptosis^39^. Although the exact role of this complex in plant PCD is unknown, the FKBP/ATP synthase subunit could participate in activation of caspase-3-like activity through a mitochondrial pathway rather than an ER pathway. This hypothesis needs further investigation to clarify the role of this complex in plant PCD. We are also aware of that in the model in Fig. 7, the mechanism by which GRP94 and Gas2 in the ER interacted with cytosolic HSP40 and caspase activity is still lacking. One possible explanation is that under severe ER stress, GRP94 participated in the retro-translocation of Gas2 from the ER to the cytosol, through ERAD pathway^40^. As a result, Gas2 might be cleaved by caspase-3-like activity at the cytoplasm side of ER membrane. However, the precise events need to be elucidated in the near future.

## Methods

### Plant materials and treatments

*Oryza sativa* L. cv. Nipponbare seedlings were grown on liquid MS medium at 25 °C using a 16 h light/8 h dark cycle. For stress treatments, 8-day-old seedlings were incubated at 4 °C on plates or in liquid MS medium containing 150 mM NaCl or 40% PEG-6000 for different amounts of time. For DTT-induced ER stress, protoplasts from cell suspensions^32^ were incubated with liquid MS medium containing 6.4 mM DTT for 18 h at 26-28 °C and then washed with MS medium two times. An equal volume of water was added to the medium without DTT as a negative control^41^.

### Purification and identification of caspase-3-like activity-related proteins

In order to purify the caspase-3-like activity-related proteins, 3–4 d rice cell suspensions^32^ were treated at 48 °C for 15 min, and then were allowed to recover at 28 °C. 300 g (fresh weight) of treated cells were collected and then ground in liquid nitrogen. Proteins from suspension cells flour were extracted for 15 min with 500 mL of extraction buffer (100 mM HEPES (pH 7.2) 10% (w/v) sucrose, 0.1% (w/v) CHAPS, 5 mM DTT and 0.01% (v/v) NP40) at 4 °C. The mixture was centrifuged at 10,000 g for 10 min and then at 100,000 g for 60 min. Total protein fraction (S-100) was collected and precipitated at 4 °C by ammonium sulfate stepwise increased concentration. The individual precipitated fractions were collected by centrifugation at 14,000 g for 30 min and dissolved in a small amount of 50 mM PBS (pH 7.5). After being dialyzed against the same buffer, the caspase-3-like activity fractions were combined and subjected to gel filtration on a Hiload 26/60 Superdex G-75 column at a flow rate of 1 mL/min, using 50 mM PBS, (pH 7.5) as the elution buffer. The absorbance of eluted fractions was measured at 280 nm. The active fractions were combined, dialyzed against 20 mM Tris/HCl (pH 8.0) at 4 °C overnight and purified further on a Mono-Q column equilibrated with 20 mM Tris/HCl buffer (pH 8.0). Proteins were eluted on a gradient of NaCl (0–1 M) in 20 mM Tris/HCl buffer (pH 8.0) at a flow rate of 1 mL/min. The active fractions were combined and de-salted with a Hitrap desalting column. In order to enrich the activity, the active fractions were incubated with 100 μM Biobinyl-DEVD-CHO overnight at 4 °C with gentle shaking, and then were loaded onto a Softlink^TM^ soft release resin column (Promega, USA). After washing the column, proteins were eluted with elution buffer (0.1 M PBS, 5 mM D-biotin (pH 7.0)) and then analyzed by LC-MS/MS directly. LC-MS/MS were conducted by a Nano LTQ Orbitrap XL mass spectrometer (Thermo Finnigan, San Jose, CA). LC–MS/MS data was acquired in data-dependent acquisition controlled by BioworksBrowser 3.3. Database searches were performed using the BioworksBrowser 3.3 program.

### Protoplast preparation

Rice suspension cells from wild-type and *GAS2 RNAi* lines were routinely propagated and cultured at 28 °C. Protoplasts were isolated from 3–4 d suspension cells according to Maas *et al*.^42^. For the protoplast isolation from green tissue, rice seedlings were grown in the nutrient solution 28 °C for 7–10 days. 10 cm high seedlings were used for protoplast isolation according to Wang *et al*.^43^.

### RNA extraction from rice seedlings

Total RNA was isolated from rice protoplasts or roots of seedlings using the Trizol reagent (Takara, Japan). Total RNA (5 μg) was used for cDNA synthesis with the PrimeScript™ RT Reagent Kit with gDNA Eraser (Takara, Japan) according to the manufacturer’s instructions.

### Real-time quantitative reverse transcription polymerase chain reaction analysis

Quantitative real-time polymerase chain reaction was conducted using a CFX 96 fluorescent quantitative PCR apparatus (Bio-Rad, Hercules, CA, USA). Gene-specific primers were used to detect the OsGAS2 transcripts and 17S rRNA was used as an internal control. Each sample was performed in three independent experiments and the primer sequences are detailed below.

*OsGAS2*-F: TTGGTAAGGAGAAGGAGTTC; *OsGAS2*-R: AGGCTGGTGGTACTATGT.

*17sRNA*-F: ACACGGGGAAACTTACCAGGTC; *17sRNA*-R: CCAGAACATCTAAGGGCATCAC.

### Construction of *RNAi-OsGAS2* cell line

To produce *RNAi-OsGAS2*, primers *Ri*1-F/*Ri*1-R for *OsGAS2* fragment 1 and primers *Ri*2-F/*Ri*2-R for *OsGAS2* fragment 2 were designed (*Kpn* I/*Sac* I sites and *Mlu* I/*BamH* I sites are shown in italics):

*Ri*1-F: GG*GGTACC*GCTGAGCATGATATGCCAGAAC; *Ri*1-R: C*GAGCTC*TCCTTCCGAACACGAGAAGC.

*Ri*2-F: CG*ACGCGT*TCCTTCCGAACACGAGAAGC; *Ri*1-R: CG*GGATCC*GCTGAGCATGATATGCCAGAAC.

The amplified fragment 1 and 2 were double digested with *Kpn* I/*Sac* I and *Mlu* I/*BamH* I, respectively, and then cloned into the *RNAi* vector LH-FAD2-1390. The recombinant plasmids were transformed into rice suspension cells by *Agrobacterium* infection.

### Subcellular localization of the OsGAS2, OsGrp94 and HSP40 protein in rice mesophyll protoplasts

For the subcellular localization study, the full length *OsGAS2* (Os01g0276800), *OSGrp94* (Os06g0716700) and putative HSP40 (Os02g0100300) cDNA were amplified using gene-specific primers (*BamH* I and *Kpn* I site are shown in italics):

*OsGAS2*: F-CG*GGATCC*ATGGGGCTCCACGCGATCC; R-GG*GGTACC*GCGAGTTCATCATGGTCACGCT.

*OsGrp94*: F-CG*GGATCC*ATGCGCAAGTGGGCGCTCTCC; R-GG*GGTACC*CTACAGCTCGTCCTTATCATA.

*HSP40:* F-CG*GGATCC*ATGGATGCTTCTCGCGTTGGC; R-GG*GGTACC*TTACTGGGACCCATTGAATTT.

The PCR products were confirmed by sequencing and then gel-purified with the AxyPrep DNA Gel Extraction Kit (Axygen, China). The products were then double digested with *BamH* I and *Kpn* I and cloned into the subcellular localization vector pXZP008^44^. A reporter gene encoding green fluorescent protein (GFP) was fused to *OsGAS2*, *OsGrp94* and *OsHSP40*, which is driven by the cauliflower mosaic virus 35S promoter (35S: *OsGAS2*/*OsGrp94*/*OsHSP40*–*GFP*), and then transformed and transiently expressed in rice mesophyll protoplasts. After incubation for 16 h at 26 °C in the dark, transformed protoplasts were stained with 100 nM ER-Tracker™ Blue-White DPX (E12353, Molecular Probes, USA) for 30 min at 28 °C in darkness and washed twice with W5 solution. Fluorescence was visualized using a PerkinElmer UltraVIEW VoX confocal microscope. The ER was identified using E12353 as a control.

### Expression and purification of recombinant his-tagged wild type and D^110^A/D^113^A double mutant OsGAS2 protein

In order to express the recombinant OsGAS2 in *Eschericia coli*, the full length *OsGAS2* cDNA (*Os01g0276800*,WT) and D^110^A/D^113^A mutant (Mu) was amplified using gene-specific primers (*Nde* I and *EcoR* I sites are shown in italics):

WT: F-GGAATTC*CATATG*GCCTCCAGGCCGCCGCTCGA;

WT: R-G*GAATTC*TTAGAGTTCAYCATGGTCACG;

D^113^A: F- ACGGGAGTGATGAGTATGCTAGCAATGTCACTTGCAAG

D^113^A: R- CTTGCAAGTGACATTGCTAGCATACTCATCACTCCCGT

D^110^A/D^113^: F- TTGCTGCGACGGGAGTGCTGAGTATGCTAGCAATGTC

D^110^/D^113^A: R- GACATTGCTAGCATACTCAGCACTCCCGTCGCAGCAA

The sequencing-confirmed PCR products were gel-purified and double digested with *Nde* I/*EcoR* I, cloned into pET-28a with His_6_-tag, and then transformed into *E. coli* DE3. Expression was induced using 0.3 mM isopropyl-b-D-1- thiogalactopyranoside (IPTG) at 37 °C for 4 h. Cells were harvested and resuspended in lysis buffer (50 mM NaH_2_PO_4_ (pH 8.0), 300 mM NaCl). After ultrasonic disruption, the mixture was then centrifuged at 10,000 × g for 1 h at 4 °C. The resulting supernatant was loaded onto a Ni-NTA resin column (GenScript, China). After washing the column, His-tagged OsGAS2 was eluted using elution buffer (50 mM NaH_2_PO_4_ (pH 8.0), 300 mM NaCl, and 250 mM imidazole).

### OsGAS2 degradation by caspase-3-like activity *in vitro*

Purified OsGAS2-His_6_ protein (100 μg) was incubated with caspase-3 or whole cell lysate from heat-treated rice suspension cells for 5 h at 37 °C in assay buffer (50 mM HEPES-KOH (pH 7.5), 10% glycerol, 50 mM KCl, 2.5 mM MgCl_2_ and 1 mM DTT). Digested products were separated by SDS-PAGE and detected by western blotting using a monoclonal mouse anti-poly histidine antibody (1:1000 dilution, BOSTER, China) as a probe.

### Conditional expression of *OsGAS2* fragments in rice mesophyll protoplasts

*OsGAS2* fragments: N- and C-framents were amplified using gene-specific primers (*Xho*I and *Spe*I sites are shown in italics):

OsGAS2-N: F-CGGGATCCATGGCCTCCAGGCCGCCGCTC

OsGAS2-N: R-GGGGTACCAAATCATACTCATCACTCCCGTCGCAG

OsGAS2-C: F-CGGGATCCATGAGCAATGTCACTTGCAAGAATAC

OsGAS2-C: R-GGGGTACCAAGAGTTCATCATGGTCACGCTGG

The products were double digested with *Xho* I/*Spe* I, and then directionally cloned into the dexamethasone (Dex)-inducible binary vector pTA7002. The resulting constructs and the vector control (20–25 μg) were transformed into rice mesophyll protoplasts.

### Caspase-3 like activity assay

Protoplasts transformed with the pTA7002 vector control or pTA7002-N/C were resuspended in W5 medium (supplemented with 10 mM Dex) and incubated in a six-well culture plate at 28 °C in darkness for specific amounts of time. After incubation, the protoplasts were treated at 48 °C for 15 min and allowed to recover at 28 °C for 6 h. The protoplasts were suspended in lysis buffer (50 mM HEPES (pH 7.4), 100 mM NaCl, 250 mM sucrose, 0.1% CHAPS, 1 mM DTT, 0.1 mM EDTA, 1 mg/mL pepstatinin, 8 mg/mL aprotinin, 10 mg/mL leupeptin). The suspensions were homogenized on ice and centrifuged at 12,000 × g for 5 min at 4 °C, the soluble cytosolic fractions were collected and incubated in assay buffer (50 mM HEPES-KOH (pH 7.5), 10% glycerol, 50 mM KCl, 2.5 mM MgCl_2_, and 1 mM DTT) supplemented with 70 mM N-acetyl-Asp-Glu-Val-Asp-7-amino-4-methylcoumarin (Ac-DEVD-AMC, EnzoLife Sciences, USA). The reaction system was incubated without light at 30 °C for 1 h. The fluorescence was measured in a microplate reader (TECAN, USA) at 380/460 nm. The difference in fluorescence before and after incubation was normalized against the corresponding protein concentration. All assays were performed using three independent samples.

### Electron microscopy

DTT-treated protoplasts were fixed with 2.5% (w/v) glutaraldehyde in CPW-9M (pH 5.8) at 4 °C overnight and post-fixed with 1% (w/v) osmium tetroxide for 3 h at room temperature. After staining with 1% (w/v) uranyl acetate at room temperature for 2 h, the protoplasts were dehydrated using several different mixtures of ethanol, water, and propylene oxide, and then embedded with Spurr’s resin. Sections measuring ~70 nm thick were cut and stained with uranyl acetate and lead nitrate. The section samples were observed with an electron microscope (H-7650, Hitachi).

### Measurement of cytosolic Ca^2+^ content

The Ca^2+^-sensitive fluorescent dye, Fluo-3-AM was used to detect the relative change of the Ca^2+^-dependent fluorescence in protoplasts. Protoplasts were incubated with 5 μM preheated Fluo-3-AM (Dojindo Laboratories, Japan) for 30 min at 37 °C, as specified by the manufacturer’s instructions. Then the cells were washed twice and re-suspended in HBSS solution containing 0.4 M mannitol. The Fluo-3 fluorescence was measured with a PerkinElmer UltraVIEW VoX confocal microscope using excitation and emission wavelengths of 488 and 525 nm, respectively. The fluorescence intensity of the protoplasts was measured by delimiting the individual protoplast, and the mean fluorescence of the protoplasts was measured and analyzed with Image Pro Plus. 8–10 protoplasts were included for each treatment.

### LSDs labeling

Protoplasts treated with DTT and/or 3-MA were collected and incubated with 50 nM LysoTracker Red DND-99 (Molecular Probes, USA) for 20 min at 37 °C, washed with CPW-9M buffer three times, and then examined by a PerkinElmer Ultra VIEW VoX confocal microscope. The wavelength of excitation was 561 nm and the emission signals were measured at 590 nm.

## Additional Information

**How to cite this article**: Cui, J. *et al*. Glucosidase II β-subunit, a novel substrate for caspase-3-like activity in rice, plays as a molecular switch between autophagy and programmed cell death. *Sci. Rep.* **6**, 31764; doi: 10.1038/srep31764 (2016).

## Figures and Tables

**Table 1:** LC-MS/MS analysis of peak I and II in Fig. 1.

Accession	Protein name	Mw	PepCount	Sequence	UniquePepCount	Coverage(%)
Os01g0276800	Glucosidase 2 beta subunit [Oryza sativa Japonica Group]	69316.69	9	K.ASQVEGHSTTSLGR.W	6	15.15
				K.ASQVEGHSTTSLGR.W		
				K.LIEKAEEEER.L		
				K.VAFAKDEAELAK.L		
				R.DKLNDDFCDCPDGTDEPGTSACPEGK.F		
				R.DKLNDDFCDCPDGTDEPGTSACPEGK.F		
				R.ISTLTDKLK.H		
				R.VNDGICDCCDGSDEYDSNVTCK.N		
				R.VNDGICDCCDGSDEYDSNVTCK.N		
Os06g0716700	Glucose-regulated protein 94 [Oryza sativa Japonica Group]	93045.01	4	K.IM*QSQTLSDASK.Q	2	2.71
				K.IM*QSQTLSDASK.Q		
				K.IM*QSQTLSDASK.Q		
				K.LGIIEDATNR.N		
Os07g0188800	Methylmalonate-semialdehyde dehydrogenase [Oryza sativa Japonica Group]	56980.21	14	K.AESLNDAIQIVNR.N	7	15.79
				K.AESLNDAIQIVNR.N		
				K.AGVQFFTQIK.T		
				K.AGVQFFTQIK.T		
				K.ASFAGDLNFYGK.A		
				K.ASFAGDLNFYGK.A		
				K.LAENITTEQGK.T		
				K.LAENITTEQGK.T		
				K.LAENITTEQGK.T		
				K.LIQSGADNGAR.V		
				K.LIQSGADNGAR.V		
				R.ANM*DKLAENITTEQGK.T		
				R.ASSLVVNSGM*ASDADLGPVISK.Q		
				R.ASSLVVNSGM*ASDADLGPVISK.Q		
Os08g0525600	Peptidyl prolyl cis trans isomerase [Oryza sativa Japonica Group]	64121.75	10	K.DGFFCPALAK.A	6	9.83
				K.LGQGQVIK.G		
				K.TVTEIGDDKK.I		
				K.TVTEIGDDKK.I		
				K.VGEEKEIGK.Q		
				K.VGEEKEIGK.Q		
				K.VTCNLNNAACK.L		
				R.LEDGTVISK.S		
				R.LEDGTVISK.S		
				R.LEDGTVISK.S		
Os05g0553000	ATP synthase beta subunit [Oryza sativa Indica Group]	58934.22	3	K.IGLFGGAGVGK.T	3	6.34
				K.VVDLLAPYQR.G		
				R.FTQANSEVSALLGR.I		
Os02g0100300	Putative tetratricopeptide repeat (TPR)-containing protein, HSP40 [Oryza sativa Japonica Group]	44151.6	12	K.AILLNPLSAIM*YGTR.A		
				K.AILLNPLSAIM*YGTR.A	6	13.24
				K.AILLNPLSAIMYGTR.A		
				K.M*GDPSIDVTEENR.D		
				R.AEAQAAYDKAK.R		
				R.AEAQAAYDKAK.R		
				R.DANAALEINPDSAK.G		
				R.DANAALEINPDSAK.G		
				R.DANAALEINPDSAK.G		
				R.RAEAQAAYDK.A		
				R.RAEAQAAYDK.A		
				R.RAEAQAAYDKAK.R		
